# 

**Published:** 1898-10

**Authors:** 


					﻿ESTABLISHED I85S.	SUBSCRIPTION, >2.00.
ATLANTA
IMGftHflD SMfiOn
1	1	SURGEON GENERAL’S OFFICE
JOURNAL. OCT.-8-1898
..................:..--I	■'
newuse^e8 Atlanta, Ga., October, 1898.	No. 8.
				

